# Study on the Reaction Kinetics of Sulfur Mustard, Nitrogen Mustard and Their Chosen Analogues with Sodium Ethoxide

**DOI:** 10.3390/molecules30040780

**Published:** 2025-02-07

**Authors:** Klaudia Kozon, Jakub Nawała, Paweł Sura, Stanisław Popiel

**Affiliations:** Institute of Chemistry, Military University of Technology, Kaliskiego 2, 00-908 Warsaw, Poland; klaudia.kozon@wat.edu.pl (K.K.); jakub.nawala@wat.edu.pl (J.N.); pawel.sura@wat.edu.pl (P.S.)

**Keywords:** chemical warfare agents, sulfur mustard, nitrogen mustard, chemical warfare agent analogues, decontamination, reaction kinetics

## Abstract

The course and kinetics of the reactions of sulfur mustard, nitrogen mustard and their selected analogues with sodium ethoxide were studied using a gas chromatograph coupled with a mass spectrometer. 2-chloroethyl ethyl sulfide (CEES), a monofunctional analogue of sulfur mustard (HD), bis(2-chloroethyl) ether (BCEE), an oxygen analogue of sulfur mustard, and bis(2-chloroethyl)amine, an analogue of nitrogen mustard HN-3, in which one hydrogen atom remains unsubstituted with a chloroethyl group, were used as imitators of mustards. For the study, the last mentioned compound was given the acronym HN-0. The research included checking how the form of sodium ethoxide influences the reaction rate. Two solutions were used: sodium ethoxide solution obtained by dissolving a commercially available compound in crystalline form and ethoxide solution obtained by dissolving sodium in ethanol. Additionally, the extent to which diethylenetriamine (DETA) accelerates the reactions of the studied compounds with sodium ethoxide was checked. The decontamination reactions were carried out in an anhydrous environment at a constant temperature of 25.0 °C. The rate of the mustard decontamination reaction increased significantly in systems containing DETA. Therefore, this amine can be used as a catalyst for this reaction. DETA has the most significant effect on the rate of the reaction of sodium ethoxide with CEES. The effect of the EtONa form was tested in the decontamination reaction of HD, revealing that both forms are equally effective, with only minor differences in reaction rates. Freshly synthesised sodium ethoxide reacts with HD 1.24 times faster. The study also assessed whether selected non-CWA compounds can be successfully used in studies as mustard imitators. Nitrogen mustard and bis(2-chloroethyl)amine reactions proceed according to the same mechanism—nucleophilic substitution. Bis(2-chloroethyl)amine reacts slightly faster than HN-3, both in solution with and without the addition of a catalyst. Sulfur mustard (HD) and CEES with sodium ethoxide and DETA undergo an elimination reaction, while BCEE undergoes a substitution reaction, which proceeds much slower. The observed differences disqualify BCEE as a sulfur mustard imitator. HD and CEES react with sodium ethoxide and DETA so quickly that the exact kinetic parameters under the developed experimental conditions could not be determined.

## 1. Introduction

Chemical warfare agent (CWA) is a term used to describe chemical compounds with appropriate physicochemical and toxic properties that make them suitable for use as chemical weapons of mass destruction. These are, therefore, compounds used during wars or terrorist attacks, causing death or loss of health for many people. Initially, natural toxins of plant and animal origin were used. With the development of science, toxic compounds began to be synthesised, and today, they can be divided into seven groups: blood agents, lung-damaging agents, nerve agents, vesicants, lacrimators, vomiting agents and incapacitating psychochemicals [1]. Chemical weapons were used on a large scale for the first time during World War I [2,3]. The devastation led to the 1925 Geneva Protocol “For the Prohibition of the Use in War of Asphyxiating, Poisonous or Other Gases, and of Bacteriological Methods of Warfare”; however, it did not cover the production of chemical warfare agents, and they started to reappear in armed conflicts soon after. During World War II, the countries with the largest stockpiles of CWAs were Great Britain, Germany, the USA, the USSR and Japan [4].

In 1997, the Chemical Weapons Convention (CWC) came into force, aiming to prohibit the development, production and use of chemical weapons [5]. However, chemical warfare agents remain a concern as some nations and terrorist groups violate the treaty. Another issue is the chemical weapons dumped in seas after World War I and World War II [4,6,7], including sulfur mustard, which is difficult to neutralise and poses a serious environmental risk. It makes sense to develop new decontamination technologies because there are no perfect decontaminants, and each of them has its advantages and disadvantages. To progress in this research, understanding the reaction mechanisms and identification of the products formed in previously developed methods is necessary [8]. To prevent treaty violations, address these threats and prepare for potential future use of chemical warfare agents, effective methods for detecting, analysing, and neutralising them are crucial.

Bis(2-chloroethyl) sulfide (HD)—is the primary representative of CW agents from the group of vesicants and one of the earliest chemical warfare agents used for military purposes [8]. It is the most decontamination-resistant compound among vesicants, so new decontamination methods are commonly developed based on its example. Although HD is not one of the most lethal CWAs, it poses many threats to humans and other living organisms. Its oily consistency and very low solubility in water give it extraordinary environmental durability [3,9]. Sulfur mustard (SM) has been the most widely used chemical warfare agent in the last century. SM continues to pose serious health problems for people exposed to it [10].

The group of nitrogen mustards includes three compounds, N-ethyl bis(2-chloroethyl)amine (HN-1), N-methyl bis-(2-chloroethyl)amine (HN-2) and tris(2-chloroethyl)amine (HN-3). All three compounds are oily liquids with limited solubility in water and good solubility in organic solvents. Nitrogen mustards are highly toxic compounds strictly controlled by the Chemical Weapons Convention. HN-3 is practically insoluble in water. In an aqueous environment, nitrogen mustard, like sulfur mustard, undergoes a hydrolysis reaction, yet it is much slower. The most stable of the three mustards is HN-3, and its vesicant properties are comparable to the properties of HD [11,12,13]. Sulfur and nitrogen mustards are environmentally persistent, especially at lowered temperatures, where they can freeze while retaining their vesicant properties. Both liquid and vapour forms of mustard readily penetrate ordinary clothing, posing prolonged risks in contaminated areas. The decontamination of sulfur and nitrogen mustards poses significant challenges due to their chemical properties, persistence in the environment and health risks during the decontamination process [14].

Nowadays, several methods of decontaminating CWAs are present, depending on the toxic agent and the type of surface that came into contact with it. Chemical warfare agents can undergo hydrolysis, oxidation or react with alkoxides. Many types of decontaminants are available on the market, but there is still no ideal, all-purpose method that meets all the required criteria. The main goal of research into CWA decomposition is to design a method that will primarily allow for the fastest and most effective decomposition of a given toxic agent, creating products that do not have toxic properties [15,16,17]. Finding non-toxic compounds similar in molecular structure and reacting according to the same mechanisms as CWAs and ensuring the safety of the person performing the tests is very helpful in CWA studies. In this study, three compounds not classified as CWAs were examined, with a similar structure to sulfur mustard and nitrogen mustard HN-3, respectively.

Bis(2-chloroethyl) ether [BCEE] and 2-chloroethyl ethyl sulfide are compounds selected as sulfur mustard simulants. BCEE is the molecule that differs from HD in the central atom—in HD, the central atom is sulfur, and in BCEE, oxygen. CEES respectively differs from HD in that it replaces a chlorine atom with a hydrogen atom on one chloroethyl branch. The compound with a similar structure to HN-3 selected for the discussed studies is bis(2-chloroethyl)amine. It is a secondary amine, composed of only two 2-chloroethyl groups instead of three as in HN-3. Based on the reactions, it was possible to assess to what extent these compounds imitate the chemical properties of the corresponding CW agents.

Many CWA decontaminants are available on the market, including organic decontaminants. They are based on different reactions. Organic decontaminants usually contain alcoholates, amino alcoholates or other compounds with similar effects. The composition of the DS-2, used by the US Army, includes sodium hydroxide (2%), 2-methoxyethanol (28%) and diethylenetriamine or another amine, constituting 70% of the entire mixture [15]. The active substance in DS-2 is 2-methoxyethanol, a nucleophile that converts CWAs into non-toxic products. The composition of the Polish ORO decontaminant includes sodium ethoxide, ethanol and two amines: monoethanolamine (MEA) and diethylenetriamine (DETA). The active ingredients of ORO, which directly participate in the decontamination reaction, are sodium ethoxide and sodium aminoethoxide. The second decontaminant—C9—consists of sodium, monoethanolamine and 2-ethoxyethanol [18,19,20,21,22]. Since the action of organic decontaminants is based on the reaction of CWA with alcoholates, it was decided to use pure sodium ethoxide (as a purchased solid substance or freshly synthesised in the laboratory) for the discussed studies without auxiliary components contained in ready-made decontamination solutions. The studies also examine the impact of adding diethylenetriamine (DETA), the primary component (by weight) of DS-2 and ORO decontaminants, on the decontamination process of chemical warfare agents (CWAs). Comparing the results will help determine whether DETA effectively catalyses the reactions with the tested compounds.

## 2. Results and Discussion

### 2.1. Reactions of the Studied Compounds with Alcoholic Sodium Ethoxide Solution

**Figure 1:**
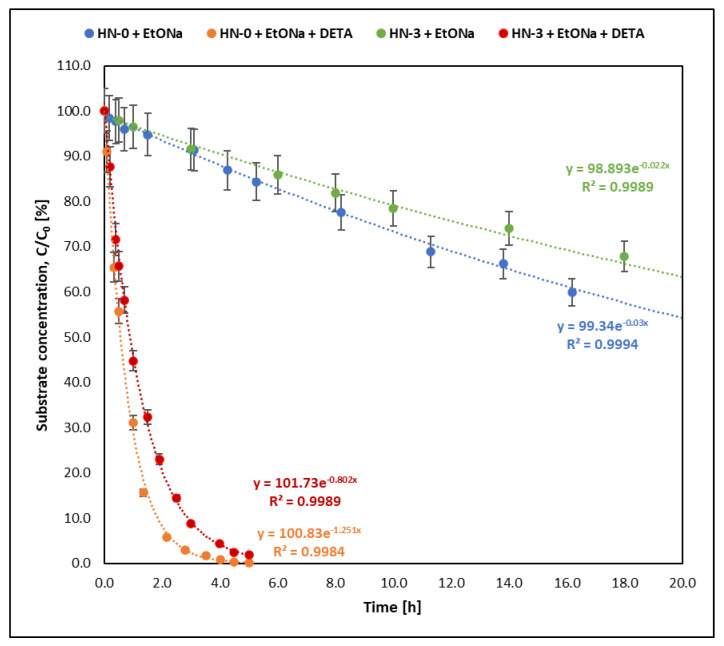
Reaction kinetics of nitrogen mustard HN-3 and its analogue HN-0 with sodium ethoxide with and without amine (DETA).

**Figure 2:**
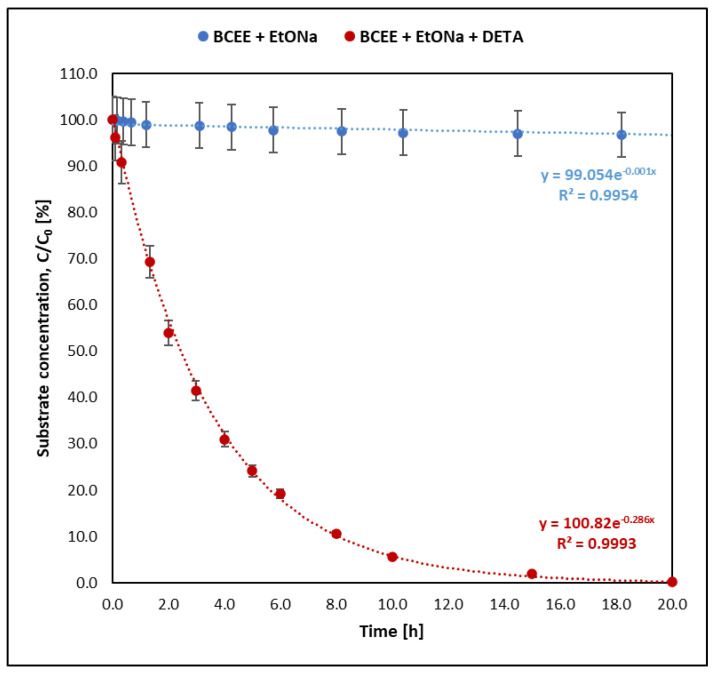
Reaction kinetics of BCEE with sodium ethoxide with and without amine (DETA).

**Figure 3:**
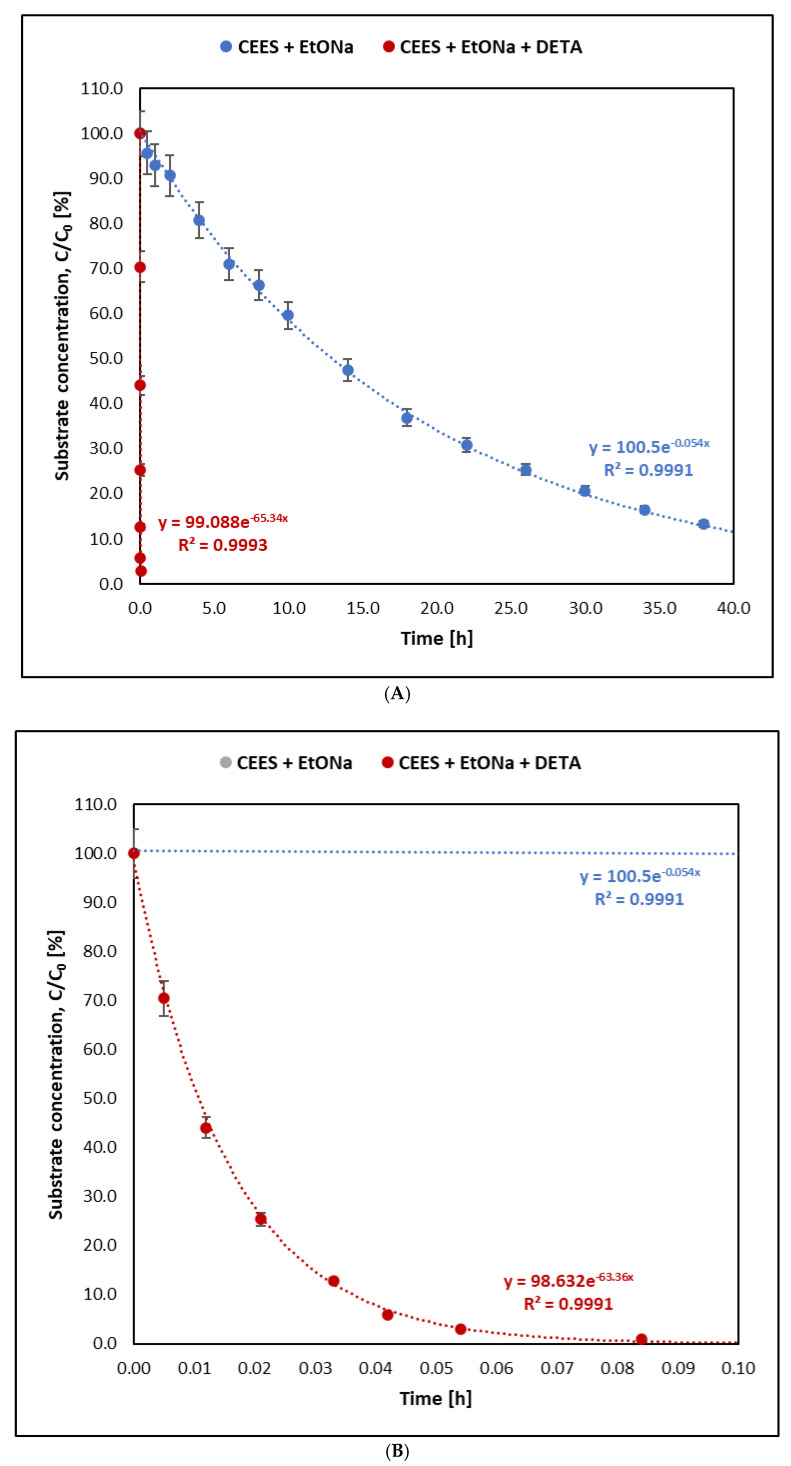
Reaction kinetics of CEES with sodium ethoxide with and without amine (DETA): (**A**) time range 0–40 h; (**B**) time range 0–0.05 h.

**Figure 4:**
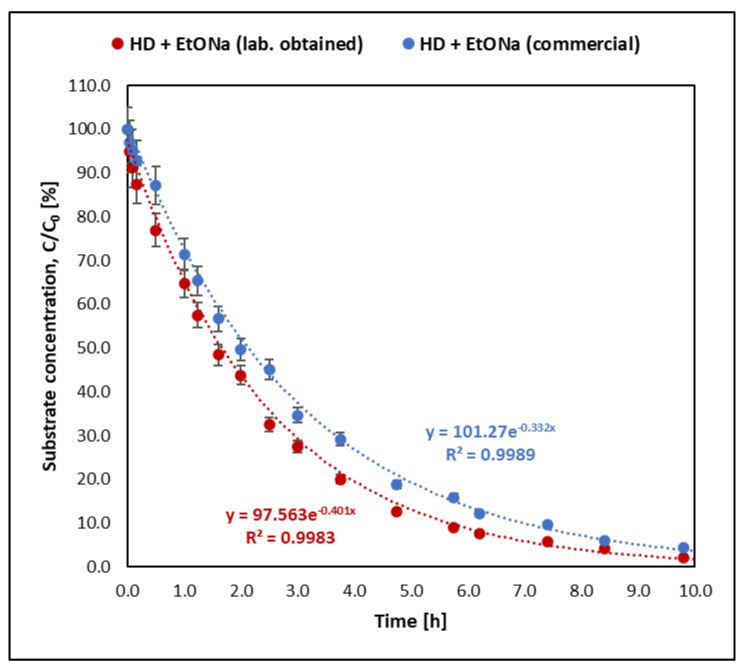
Kinetics of the reaction of sulfur mustard with sodium ethoxide obtained by dissolving purchased sodium ethoxide in ethanol and in a sodium ethoxide solution synthesised in the laboratory from metallic sodium and ethanol. In both cases, the same concentration of sodium ethanoate was used.

The reactions of the studied mustards and their selected analogues with sodium ethoxide proceed at very different rates. In each case, however, using sodium ethoxide and diethylenetriamine decontamination solution significantly reduced the reaction time. The change in the concentration of individual substrates during the decontamination reaction is shown in Figure 1, Figure 2, Figure 3 and Figure 4. Figure 4 compares the kinetics of the reaction of sulfur mustard with a sodium ethoxide solution prepared by dissolving a weighed amount of commercially available sodium ethoxide in ethanol and with an ethoxide solution prepared by dissolving sodium in ethanol in the laboratory on the day of the analyses.

**Figure 5:**
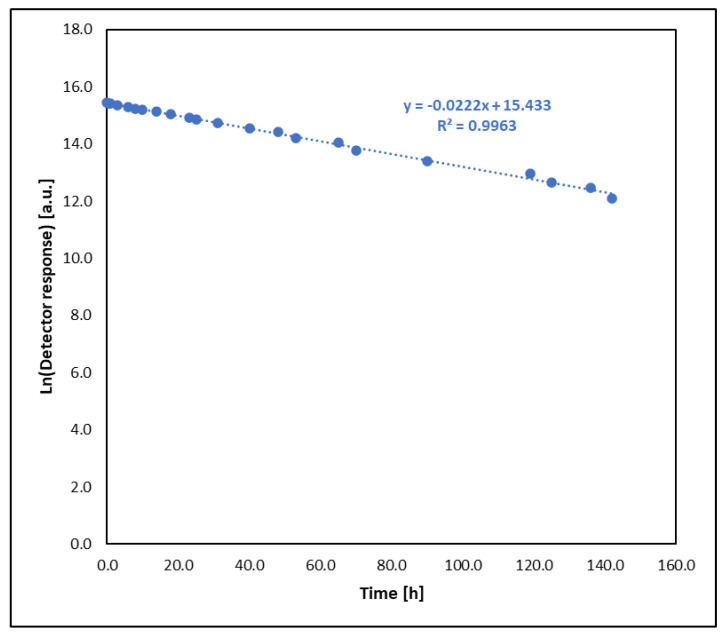
Logarithmic dependence of the detector response as a function of time for the reaction of HN-3 with sodium ethoxide.

Using the graphical method and graphs of the change in the amount of the tested substances (Figure 1, Figure 2, Figure 3 and Figure 4) and their logarithmic presentation (Figure 5), it can be stated that the reactions tested are first-order reactions. On this basis, the relationship $t_{1/2}=\frac{ln(2)}{k}\;$ can be used to determine the half-life of the tested substances.

In the decontamination reaction of the tested mustards and their analogues, sulfur mustard decomposes the fastest. The half-life of mustard in the reaction with sodium ethoxide alone (without DETA) at 25 °C was 125 min, while when the decontamination solution was a mixture of sodium ethoxide and amine, the reaction was over 120 times faster. Of the samples taken from the reaction mixture within 1.5 min from the start of the reaction, only the first one still contained sulfur mustard in an amount that allowed its detection by the given method (Figure 5). Nitrogen mustard HN-3 and its selected analogue bis(2-chloroethyl)amine react at a similar rate. The half-life of HN-3 was 30.14 h, and that of bis(2-chloroethyl)amine was 22.73 h. When using the mixture containing DETA, the half-life of both compounds was shorter than 1 h. The reactions of BCEE with sodium ethoxide, both with and without the addition of amine, were the slowest compared to the reaction rates of both mustards. In the reaction with sodium ethoxide, the half-life of bis(2-chloroethyl) ether was 533 h, and in the reaction with the decontaminant containing sodium ethoxide and amine, it was about 2.5 h.

To check whether the form of sodium ethoxide used for the study affected the reaction kinetics, sulfur mustard was also subjected to a reaction with EtONa synthesised on the same day by dissolving the appropriate amount of sodium in ethanol (Figure 3). In the case of sodium ethoxide obtained as a result of the reaction of sodium with ethanol, the decontamination of HD was slightly faster, with a half-time of 1.69 h, but these are insignificant differences. The values of the substrate half-life times and the observed reaction rate constants for all tested compounds are presented in Table 1.

### 2.2. Identification of Reaction Products

Chromatographic analysis of samples taken during each reaction allowed for the observation of how fast the substrate concentration in the sample decreased and how fast the share of intermediate and final products increased. The compound peaks appearing in the chromatograms were identified by coupling the gas chromatograph with a mass spectrometer. The obtained mass spectra were compared with the NIST library and allowed for the determination of the mechanisms by which every reaction proceeds and the resulting products. The match factors and reserve match factors for all identified compounds were above 900.

**Figure 6:**
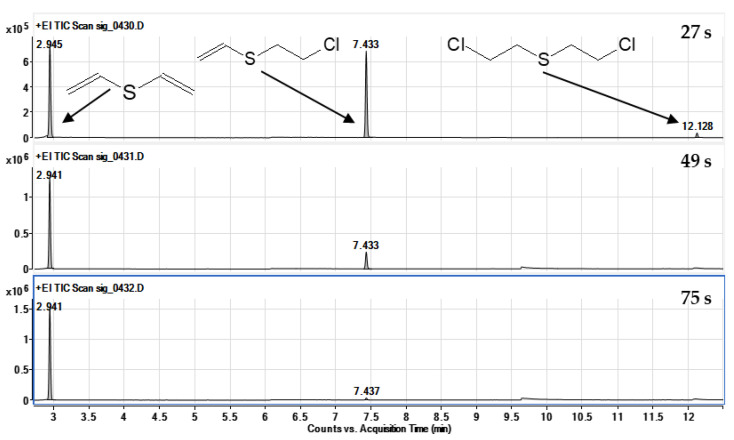
Chromatograms obtained by analysis of three samples taken during the reaction of sulfur mustard with sodium ethoxide in the presence of DETA. Visible peaks of divinyl sulfide (1), vinyl-2-chloroethyl sulfide (2) and sulfur mustard (3).

The reaction of sulfur mustard with sodium ethoxide proceeded according to the elimination mechanism. Double dehydrohalogenation occurred, and the final product of the reaction was divinyl sulfide. The reaction of mustard with the mixture of ethoxide and amine proceeded more than 120 times faster than in the case of ethoxide without adding amine. Of the samples taken from the reaction mixture within 1.5 min from the start, only the first one still contained sulfur mustard in an amount that allowed its detection by the developed method, as shown in Figure 6. The chromatograms of the remaining samples showed the intermediate product (single elimination effect) and the final product (divinyl sulfide, DVS) in various amounts. Therefore, it was impossible to determine the kinetic parameters for this reaction at a given temperature; it can only be stated that the half-time of mustard disappearance under these conditions was much shorter, 1 min.

**Figure 7:**
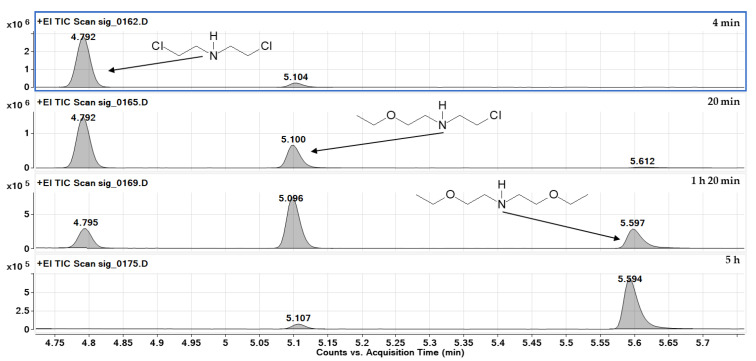
Chromatograms obtained by analysing four samples taken at different times of the reaction of HN-0 with EtONa in the presence of DETA. Visible signals of HN-0 (1), intermediate product (2) and the final product—bis(2-ethoxyethyl)amine (3).

The reaction of CEES with sodium ethoxide and DETA addition took place according to the same mechanism as HD. Dehydrohalogenation occurred on chloroethyl bound, which resulted in the creation of vinyl ethyl sulfide as a final product. The half-life of the tested substance was approximately 41 s. Completely different kinetics and mechanisms had a reaction without the addition of amine. The reaction speed value was reduced by almost 1100 times, which resulted in a 12.5 h half-life. Moreover, the mechanism proceeded according to the substitution reaction. The mass spectra of the final product, ethoxyethyl ethyl sulfide, with the interpretation of individual fragments of the spectrum, are shown in Figure 7.

Nitrogen mustard HN-3 and its selected analogue, bis(2-chloroethyl)amine, reacted at similar rates. Both compounds reacted with sodium ethoxide by the nucleophilic substitution mechanism, and the shorter reaction time of HN-0 was due to the lower order of this amine. The final product of the HN-3 reaction was tris(2-ethoxyethyl)amine, and in the HN-0 reaction, bis(2-ethoxyethyl)amine, respectively.

The compound chosen for comparison with sulfur mustard, bis(2-chloroethyl)ether, reacted with sodium ethoxide according to the nucleophilic substitution mechanism. This was the slowest reaction of all those studied in this article. The final product was bis(2-ethoxyethyl)ether.

The post-reaction solutions were observed for 30 days to see whether any other decomposition products were formed. Based on the obtained results, it can be concluded that no other compounds were formed during this time.

### 2.3. Influence of Solvent Polarity

The potential influence of ethanol polarity and other solvent properties on the kinetics and mechanisms of the reaction can be explained by taking into account several issues:

1. Ethanol is a polar protic solvent, so it can form hydrogen bonds; these bonds can solvate cations present in solution, such as the sodium cation derived from sodium ethoxide; in the elimination reaction, which is dominant in the presence of a strong base such as EtONa, ethanol can stabilise intermediate products.
2. Sodium ethoxide in an ethanol environment acts as a strong base, which causes the simultaneous elimination of a proton and a leaving group (in this case, chloride anion) with the formation of a double bond. This type of reaction proceeds better in the presence of an aprotic solvent, but taking into account the studies performed, ethanol is also a suitable solvent. The reaction rate in the case of ethanol is, however, slower.
3. Ethanol, as a protic solvent, can partially solvate the ethanoate ion (anion–proton interaction), reducing the anion’s basicity and thus reducing the reaction rate. Despite this, C_2_H_5_O- is a strong enough base to abstract a proton from the molecule and promote the elimination mechanism. In some cases (e.g., for CEES), the elimination reaction is slow enough that the competing substitution reaction plays the primary role. In aprotic solvents, the reaction rate for sodium ethanoate is much higher [23].

### 2.4. Reaction Mechanism

A proton is abstracted from the carbon atom adjacent to the carbon bonded to the chlorine atom only when the molecule adopts an s-trans (anti-periplanar) conformation relative to the leaving group. In this conformation, the bonding orbitals of the hydrogen atom and the leaving group are correctly aligned, allowing for effective orbital overlap. This circumstance facilitates the formation of a partial double bond, stabilising the transition state by reducing its energy. In the transition state, the axes of the p orbitals forming the new double bond must lie in the same plane as the axes of the sp³ orbitals of the hydrogen atom and the leaving group to ensure effective orbital interactions [23].

## 3. Materials and Methods

### 3.1. Reagents

The following reagents were used: distilled water (Milli-Q SQ 200P Purification System); sodium ethoxide (EtONa) from Sigma-Aldrich, Saint Louis, MO, USA; dichloromethane (DCM), 99.8%, ethanol, 99.8%, sodium from POCH, Gliwice, Poland; anhydrous magnesium sulfate (MgSO_4_), sodium hydroxide (NaOH), sodium chloride (NaCl), DETA from Chempur, Piekary Śląskie, Poland; bis(2-chloroethyl) ether (BCEE) and 2-chloroethyl ethyl sulfide from Merck, Darmstadt, Germany; bis(2-chloroethyl)amine from Acros Organics, Karlsruhe, Germany. Sulfur and nitrogen mustard, of purity > 98%, were obtained from the Institute of Chemistry, WAT laboratory. The purity of sulfur and nitrogen mustard was checked by gas chromatography coupled with mass spectrometry (GC-MS).

The physicochemical parameters of the used chemical warfare agents and their analogues are presented in Table 2.

### 3.2. Instrumentation

The decomposition rate of selected compounds was studied quantitatively by checking the content of samples taken from the reaction mixture at specific time intervals. A 7890A gas chromatograph coupled with a 7000 GC-MS/MS triple quadrupole tandem mass detector from Agilent Technologies (Santa Clara, CA, USA), computer-controlled by the MassHunter (B.07.06.2704, Agilent Technologies, Santa Clara, CA, USA) programme, was used for quantitative and qualitative analyses of reaction mixture. The decontamination reactions were carried out under constant temperature conditions. A water bath with a Huber Pilot ONE thermostat was used, which allowed the reaction temperature to be controlled with an accuracy of ±0.02 °C.

### 3.3. Chromatographic Analysis Conditions

The GC-MS/MS analysis was performed using a HP-5 capillary column (30 m × 0.25 mm × 0.25 μm) from Agilent Technologies. The helium carrier gas flow rate was 1 mL/min. The injector temperature was 270 °C. The temperatures of the transfer line, ion source and quadrupoles were 250, 230 and 150 °C, respectively. During the analysis, the injector was in split mode 200:1. The mass spectrometer operated in electron impact (EI) ionisation mode at 70 eV. The detector operated at full scan mode with mass range from m/z 40 to 500 and scan time 180 ms. The analysis of sulfur mustard decontamination products was carried out using the following temperature programme: the column was heated for 3 min at 40 °C, then heated up to 270 °C at a rate of 10 °C/min, and the final temperature was maintained for 1 min. The analysis of other compounds was carried out using the following temperature programme: the column was heated from 70 to 270 °C at a rate of 10 °C/min, and the final temperature was maintained for 1 min.

### 3.4. Bis(2-chloroethyl)amine Synthesis

**Figure 8:**
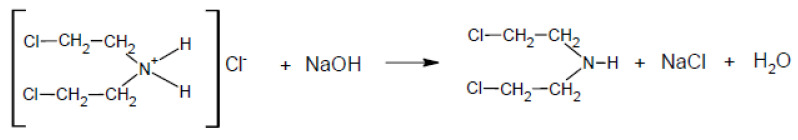
Reaction scheme for bis(2-Chloroethyl)amine synthesis.

A total of 1 g of bis(2-chloroethyl)amine hydrochloride was placed in a screw-cap test tube and dissolved in 5 mL of distilled water. Then, 2.25 mL (calculated stoichiometric amount with a slight excess) of previously prepared 10% sodium hydroxide solution was added to the solution. The synthesis reaction proceeded according to the scheme in Figure 8.

The alkaline pH was confirmed using an indicator paper. The test tube was screw-caped, and its contents were shaken for 3 min in a shaker and then centrifuged for 5 min at 2500 rpm. After centrifugation, two phases were visible in the test tube—the lower one was pure bis(2-chloroethyl)amine. The amine was separated from the aqueous phase with a pipette and transferred to a clean test tube. The remaining water was removed by adding anhydrous magnesium sulfate. The synthesis reaction of bis(2-chloroethyl)amine was carried out before each use.

### 3.5. Tris(2-chloroethyl)amine Synthesis

**Figure 9:**
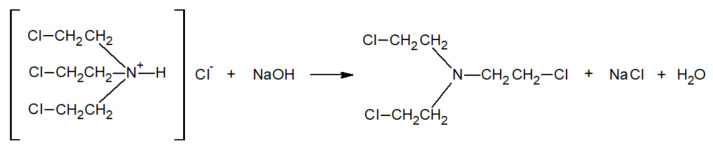
Reaction scheme for tris(2-Chloroethyl)amine synthesis.

In total, 1 g of tris(2-chloroethyl)amine hydrochloride was placed in a screw-cap test tube and dissolved in 5 mL of distilled water. Following this, 1.60 mL (slight excess) of 10% NaOH solution was added to the solution, and the basic pH was confirmed using indicator paper. The synthesis reaction proceeded according to the scheme in Figure 9.

The screw-cap test tube was shaken and then centrifuged, obtaining pure nitrogen mustard (HN-3) at the bottom. The compound was separated from the aqueous phase and dried with anhydrous magnesium sulfate, which was later separated from the amine by centrifugation. The synthesis was carried out once, and the obtained and purified HN-3 was stored in a closed test tube in a freezer.

### 3.6. Sodium Ethoxide Synthesis

In a 250 mL round-bottom flask equipped with a reflux condenser and a dropping funnel, 5 g (0.217 mol) of sodium metal was placed; 100 mL of cold ethyl alcohol was then placed in a dropping funnel. Ethanol was slowly added to the flask, and the contents were constantly and slowly stirred with a magnetic stirrer until the metal fully dissolved. After the reaction was complete, the contents of the flask were cooled. A solution of ready crystalline 3.70 g (0.0545 mol) of sodium ethoxide in 25 mL of ethanol was also prepared for comparison.

### 3.7. Decontamination Mixture Preparation

In 60 mL glass vials, 25 mL of the prepared sodium ethoxide solution in alcohol, a magnetic dipole and, depending on the reaction variant, either 25 mL of ethyl alcohol or 25 mL of diethylenetriamine (DETA) was placed. The mixtures prepared this way were placed on a magnetic stirrer in a thermostated water bath at 25 °C. The contents of the vials were stirred at 350 rpm. In order to start the decontamination reaction, 100 µL of sulfur or nitrogen mustard or one of the two simulants was added to the prepared mixture. The initial concentration of the tested substance in the decontaminant was 2 µL/mL. The reaction time measurement was started when 100 µL of the CWA (or its analogue) was placed into the vial with the decontamination mixture. All reactions were repeated three times to check the correctness of the experiment and determine errors.

### 3.8. Sample Preparation

Ten screw-capped test tubes for liquid–liquid extraction were prepared for each reaction kinetics experiment. To increase the extraction efficiency, 0.2 g of NaCl was weighed into the extraction tubes, and then 4 mL of distilled water and 2 mL of dichloromethane were added. At specified time intervals, 2 mL of liquid was pipetted from the reaction mixture and transferred to a prepared extraction tube. The contents of the test tube were shaken manually for 30 s, then centrifuged for 5 min at 2500 rpm. Then, the organic phase was transferred to another test tube containing approximately 0.2 g of anhydrous magnesium sulfate. Then, the test tube was shaken for 5 min at the rate of 1500 shakes per minute, which allowed the removal of the trace amounts of water from the organic phase. The dried solution was decanted from over the sediment of MgSO_4_ and analysed by GC-MS/MS.

## 4. Conclusions

- Adding DETA to the alcoholate significantly increases the rate of decontamination reactions for all five tested compounds. DETA acts as a catalyst regardless of the reaction mechanism, enhancing speed and effectiveness. Its most pronounced impact is observed in the reaction of sodium ethoxide with CEES, where the rate is 1100 times higher compared with reactions without DETA. It is an important additive to commercial decontaminants.
- In reactions of nitrogen mustards (HN-3 and HN-0), the dominant mechanism is nucleophilic substitution, where ethoxy groups replace chlorine atoms. HN-3 forms tris(2-ethoxyethyl)amine, while HN-0 forms bis(2-ethoxyethyl)amine, with both reactions proceeding at similar rates. Due to its similar reactivity, HN-0 can serve as a nitrogen mustard analogue for research purposes.
- Sulfur mustard (HD) reacts much faster than its analogues BCEE and CEES. BCEE reacts the slowest (t1/2 = 500 h), while HD and CEES in EtONa + DETA solutions react completely within 1 min. Reaction mechanisms vary: for HD, elimination is dominant, producing divinyl sulfide; for BCEE, nucleophilic substitution occurs, forming bis(2-ethoxyethyl) ether. Due to significant reaction rate differences, BCEE is unsuitable as a sulfur mustard analogue. CEES follows elimination in the presence of amine and substitution without it.
- The method of preparing the sodium ethoxide solution (via synthesis from sodium or by dissolving crystalline EtONa in ethanol) does not significantly influence reaction kinetics. The reaction rate differs only slightly (1.24 times faster with synthesised EtONa), confirming that both preparation methods are equally effective for decontamination.

This study highlights the role of sodium ethoxide as a chemical warfare agent (CWA) decontaminant and the catalytic role of DETA in enhancing decontamination rates. It also identifies critical reaction mechanisms and kinetic differences among chemical warfare agents and their chosen analogues. While HN-0 and CEES can be successfully used as CWA imitators, BCEE is an unsuitable choice due to the large differences in reaction rates and different reaction mechanisms. These findings underline the importance of selecting appropriate analogues for research and confirm the practicality of both EtONa preparation methods for effective use in decontamination solutions.

## Figures and Tables

**Table 1:** Half-life and observed reaction rate constants determined from the reactions of four compounds with sodium ethoxide with and without diethylenetriamine.

Tested Compound	t_1/2_ [h]		k_obs_ [h^−1^]	
	EtONa	EtONa + DETA	EtONa	EtONa + DETA
HD—sulfur mustard	1.82 ± 0.09	<0.02	0.3815 ± 0.0185	-
BCEE—sulfur mustard analogue	677.34 ± 36.79	2.44 ± 0.05	0.0010 ± 0.0001	0.284 ± 0.005
CEES—sulfur mustard analogue	12.53 ± 0.65	0.01 ± 0.00	0.0553 ± 0.0029	65.067 ± 2.091
HN-3—nitrogen mustard	31.04 ± 1.73	0.85 ± 0.02	0.0223 ± 0.0012	0.795 ± 0.018
HN-0—nitrogen mustard analogue	22.60 ± 1.51	0.55 ± 0.02	0.0307 ± 0.0021	1.251 ± 0.044

**Table 2:** List of compounds used in kinetic studies, information about them and their physicochemical properties.

Compound Parameter	Bis(2-chloroethyl) Sulfide	Bis(2-chloroethyl) Ether 2,2′-Dichlorodiethyl Ether	2-chloroethyl Ethyl Sulfide	Tris(2-chloroethyl)amine Trichlormethine	Bis(2-chloroethyl)amine
Common name	Sulfur mustard Mustard gas	Sulfur mustard analogue	Half mustard	Nitrogen mustard	Nitrogen mustard analogue
Molecular formula	C_4_H_8_Cl_2_S	C_4_H_8_Cl_2_O	C_4_H_9_ClS	C_6_H_12_Cl_3_N	C_4_H_9_Cl_2_N
Abbreviation/code	H/HD	BCEE	CEES	HN-3	HN-0
Structure	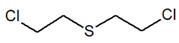	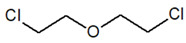	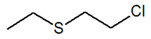	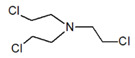	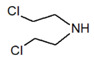
CAS number	505-60-2	111-44-4	693-07-2	555-77-1	334-22-5
Molecular weight	159.08	143.01	124.63	205.54	142.02
Boiling point (760 mm Hg)	215–217 °C	178 °C	156 °C	230–235 °C, decomp.	46–50 °C
Melting point	13–14 °C	−50 °C	−48.6 °C	−3.7 °C	Not available
Water solubility [g/L]	0.92 (22 °C)	10.2	N.A	0.16	Slightly soluble in water
Density [g/cm^3^]	1.27 (20 °C)	1.22 (20 °C)	1.0663	1.24	1.13
Vapour pressure [mm Hg (20 °C)]	0.072	0.71	3.4 (25 °C)	0.011	0.267 (25 °C)
Volatility [mg/m^3^]	610	548	16570	12	205
Log K_OW_	2.14	1.29	2.17	2.27	Not available
Van Den Dool–Kratz RI, non-polar column	1177	984	895	1411	1087
Henry’s Law Constant (H, atm × m^3^/mol)	2.1 × 10^−5^	2.9 × 10^−5^	4.9 × 10^−4^	3 × 10^−7^	Not available
LD50 Oral—Rat—[mg/kg]	2.4	75.0	252.0	5.0	1150.0 (hydrochloride)

## Data Availability

Data are made available by the authors upon request.
